# Effects of vitamin D signaling in cardiovascular disease: centrality of macrophage polarization

**DOI:** 10.3389/fcvm.2024.1388025

**Published:** 2024-06-25

**Authors:** Anton Fliri, Shama Kajiji

**Affiliations:** Emergent System Analytics LLC, Clinton, CT, United States

**Keywords:** aging, cardiovascular disease, inflammation, immunity, macrophage autophagy, nitric oxide, machine learning, vitamin D

## Abstract

Among the leading causes of natural death are cardiovascular diseases, cancer, and respiratory diseases. Factors causing illness include genetic predisposition, aging, stress, chronic inflammation, environmental factors, declining autophagy, and endocrine abnormalities including insufficient vitamin D levels. Inconclusive clinical outcomes of vitamin D supplements in cardiovascular diseases demonstrate the need to identify cause-effect relationships without bias. We employed a spectral clustering methodology capable of analyzing large diverse datasets for examining the role of vitamin D's genomic and non-genomic signaling in disease in this study. The results of this investigation showed the following: (1) vitamin D regulates multiple reciprocal feedback loops including p53, macrophage autophagy, nitric oxide, and redox-signaling; (2) these regulatory schemes are involved in over 2,000 diseases. Furthermore, the balance between genomic and non-genomic signaling by vitamin D affects autophagy regulation of macrophage polarization in tissue homeostasis. These findings provide a deeper understanding of how interactions between genomic and non-genomic signaling affect vitamin D pharmacology and offer opportunities for increasing the efficacy of vitamin D-centered treatment of cardiovascular disease and healthy lifespans.

## Introduction

Atherosclerosis is one of the prominent pathological conditions that lead to myocardial infarction and stroke. A significant risk factor for its development is chronological age (1), accelerated by epigenetic aging (2). Aging is associated with declines in cellular homeostasis causing inflammation (3–6) and organ system dysfunction (7–9), thereby resulting in the distortion of the healthy balance between inflammation and tissue homeostasis (10). Aging affects this balance in multiple ways including decreased efficiency of cellular housekeeping and tissue repair functions (11–13), which leads to the accumulation of cellular debris (14), increased endoplasmic reticulum stress (15) defective tissue homeostasis (16, 17), declining endocrine functions (18) changes in RNA compositions (19) immune senescence (20, 21), and increased inflammation (22, 23). Understanding how interactions between these systems can affect the outcomes of pharmacological studies (24), offering opportunities to slow the effects of aging (25) and prevent the development of associated diseases (26–28).

The central actors in maintaining tissue homeostasis are macrophages (29, 30), dendritic cells (31, 32), stem cells (33), and vitamin D (34–38). However, our understanding of their interaction remains incomplete, as evidenced by multiple inconclusive clinical trials seeking to halt the progression of cardiovascular disease associated with vitamin D deficiency (39–43). In previous reports, we have reported that vitamin D deficiency affects the tipping points that control network–network interactions in over 500 diseases, including atherosclerosis (44).

## Materials and methods

### Background information

*Cause–effect* relationships in complex biological network systems are determined using an information theory-based analysis methodology that is developed to ascertain information flows induced by drugs and diseases (probes) in biological networks (45). This *cause–effect* relationship-driven analysis approach identifies global network architectures mediating probe-induced information transfers within and between tissues and ascertains how probes affect network linkage (46–54). The statistical value of solutions resulting from this analysis is extremely significant because the global network architecture discovered in system-wide *cause–effect* analysis links information in large, orthogonal, and decentralized data sets. Furthermore, no single data point can distort the global network architecture imposed by the functional and structural interdependence of subunits linked in *cause–effect* relationships.

### Protein swarm generation and spectral clustering

**Figure 1:**
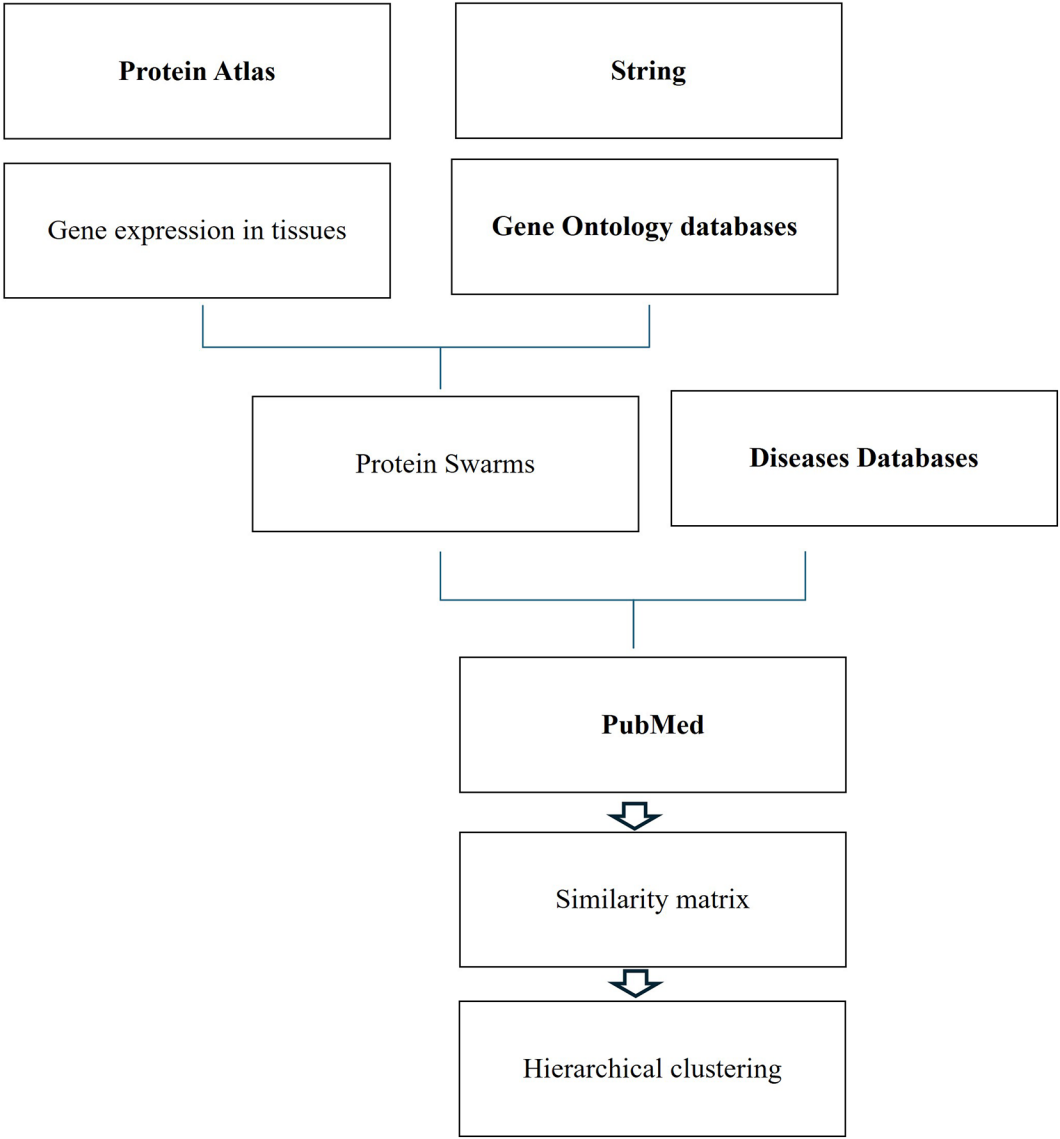
Databases and work flow used for cause effect analysis.

Functional analysis of genomic and non-genomic signaling by vitamin D in 4,821 diseases as illustrated in Figure 1 involved use of (1) the data mining algorithm developed by SystaMedic Inc. in collaboration with the University of Connecticut, (2) the databases and data analysis tools of the STRING platform (55), (3) tissue-associated protein expression data identified in the Human Protein Atlas (56), (4) information published in Medline (57), and (5) data analysis and visualization tools of Spotfire (58). Hierarchical clustering of similarity matrices obtained through Medline data extraction was used for identifying global network architectures linking probe-induced information flows in tissue–protein networks and *cause–effect* relationships (59–63).

Tissue-specific protein expression data (tissue proteomes) extracted from the Human Protein Atlas are processed using gene enrichment analysis in the STRING platform. The output of this analysis allows the identification of tissue-associated protein network fragments that contain no more than five proteins capable of interacting in a swarm-like fashion with other proteins in response to probe-induced system perturbations. Henceforth, the term protein swarms in lieu of protein network fragments is used in this analysis. Identifying and collecting protein swarms representing all tissues in the Human Protein Atlas provides descriptor sets that can be used for determining co-citation frequencies of probes and protein swarms in Medline. Viewing co-citation frequencies measurements as estimates of a probe's capacity to affect information transfers within and between protein swarms, hierarchical clustering of similarity matrices obtained through Medline data extraction identifies global network architectures linking probe-induced information flows in tissue–protein networks and cause–effect relationships.,

### Terminology

The term vitamin D herein refers to the hormone 1,25-dihydroxyvitamin D3 (calcitriol) and not its precursor, calcidiol, which is commonly used for measuring vitamin D deficiencies (64). In turn, genomic and non-genomic signaling refers to the catenation of functions resulting from (1) the binding of calcitriol at picomolar concentrations to the nuclear vitamin D receptor (VDR) (65) and (2) the binding of calcitriol at nanomolar concentrations to the membrane receptor PDIA3, also known as ERp57 and grp58 (66, 67). PDIA3 is an endoplasmic reticulum chaperone with protein disulfide isomerase activity. It regulates vitamin D genomic signaling (68); apoptosis (69, 70); efferocytosis (71), mitochondrial functions (72); functions of macrophages (73), dendritic cells (74, 75), and stem cells (76); progranulin (77); and functions of the innate and adaptive immune system (78).

## Results

### Establishment of a global effect positioning system

Identification of co-citation frequencies of 4,821 diseases with 7,371 protein swarms in Medline, construction of a 4,821 × 7,371 similarity matrix, and UPGMA hierarchical clustering of the similarity matrix using cosine as similarity measure were used to identify and select swarm clusters linked within a confidence in cluster similarity value (CCSV)of >0.92 with swarms containing vitamin D receptors (79). This process identified 29 discrete clusters containing in aggregate 656 protein swarms holding 363 proteins (disease-associated vitamin D interactome; Table 1).

**Table 1:** The proteins regulating vitamin D's genomic and non-genomic pharmacologies in 4,821 diseases isolated from 29 discrete swarm clusters containing in aggregate 363 proteins (disease-associated vitamin D interactome).

Cluster	94–98	342–348	422–433	580–596	702–709	726–730	731–745	746–779	780–788	789–795	1,055–1,061	1,255–1,264	1,519–602	1,795–1,806	1,985–1,990	2,128–2,137	2,349–2,357	2,928–2,936	2,946–3,049	3,096–3,143	3,401–3,470	3,620–3,633	3,648–3,663	3,766–3,775	4,041–4,061	4,460–4,495	5,106–5,116	6,008–6,017	7,116–7,158
	BMP7	ACTN3	C1S	AKT1	AGT	CYP27B1	BECN1	AGTR1	AGER	CFHR5	CYP24A1	AKT1	ABCG1	CFLAR	AKT1	CDK6	AKT1	AGT	AGER	AKT1	AKT1	ABCA1	ACTN3	ALDOB	ARNTL	AKAP8	AKT1	CAT	AKT1
	CYP24A1	ADRBK1	C3	CYCS	AKT1	ITGB3	BMP2	APOA1	APOA1	IL18	CYP27B1	ATG5	ACACA	EMD	CTNNB1	CFLAR	CAV1	AKT1	AGT	AXL	APCS	AKT1	AKT1	ATG5	CSF2	ARG1	ARNTL	CYP27B1	AXL
	DRD1	C1QTNF1	C4B	CYP27B1	APOA1	NFKB1	CAV1	CFL1	BECN1	IL1A	DHCR24	CAMP	AGER	INSR	EDN3	CYP27B1	CFLAR	CCL21	AKT1	BCL2L1	ARNTL	BAIAP2	BECN1	CTSD	CYP24A1	BMP4	ATG5	DHCR24	CAD
	DRD5	CAV1	CYP27B1	DRD3	ARNTL	PPARG	CCL5	CYP27B1	CAPN1	IL1B	LBR	CYP27B1	ALOX15	MAPK1	GRB2	MAPK1	CYP27B1	CX3CR1	APCS	BCL2L11	ATG5	C2	CCL5	ENO1	CYP27B1	CAPN1	CDK6	DNMT3A	CAT
	F5	CYBA	FCN2	DRD4	BMP2	SREBF2	CD58	FGF1	CASP8	IL6	TM7SF2	DHCR24	ALOX15B	PDIA3	MET	NOTCH1	DDAH1	CYP24A1	ARNTL	BTRC	AXL	CASP8	CCR3	G6PC	DRD1	CREB1	CFLAR	DNMT3B	CDK4
	ITGB3	CYP27B1	FCN3	FGB	CALR	TGFBR1	CYP27B1	GPLD1	CD40	IL6R	VDR	IRS1	ANGPTL4	SMAD2	NOTCH1	PTPN1	DDAH2	CYP27B1	ATG5	CASP7	BMP7	DLG4	CFL1	GAPDH	DRD3	CREBBP	CYBB	DRD3	CFLAR
	SOD1	DNM2	KRT1	GLUL	CCL5	TNF	EDN1	IKBKB	CD86	IRAK2		LBR	APOA1	SMAD3	NRP1	SMAD2	DNMT3B	EPCAM	BECN1	CASP8	BMPR1A	FGF2	CTF1	LIPG	DRD4	CTSD	DRD1	ECH1	CLDN1
	SOD2	EDN1	LY96	HDAC2	CYP27B1		FCN1	IL18	GRB2	LTA		MAPK1	ARNTL	TGFB1	PLA2G7	SMAD3	MAPK1	EPO	BMP2	CDK6	CD40	FZD2	CTSD	PCBP1	DRD5	FGF13	DRD3	EDN3	DDAH1
	TGFB1	EDN2	MYD88	IRS1	INS		FGF1	IL1B	IKBKB	NOS3		MTOR	ATF4		SMAD3	TCF21	NOTCH1	HHEX	BMP7	CDKN1A	CD47	MDM2	EDN1	PCBP2	GC	FOXO3	DRD4	IGF2	DDAH2
	VDR	IL1B	POU2F2	NOTCH1	NFKB1		FOXO3	IL1RN	IL33	OSMR		NOTCH1	BAIAP3		SOD2	TCF7	PROC	IDO1	BMPR1A	CFLAR	CDH2	MERTK	EDN2	PDIA3	GLUL	FYN	GLUL	INSR	DNMT3A
		KCNA1	RPS6KB1	PRKCE	S100A9		IGF1	IL2RA	NFKB1	TNF		PALM	BMP2		TCF21	TNFRSF1A	TNFRSF1A	IGF1R	CANX	CTSD	CDK4	MMP14	HAMP	PKM	IDO1	FZD1	HRAS	LBR	DNMT3B
		KCNIP1	TNFRSF13C	RAN	TNF		IL1B	IL33	PDIA3			PC	BTRC		VDR		VDR	IGF2	CASP7	CYP27B1	CDK6	NLGN1	HDAC1		IDO2	GLI3	MAX	TM7SF2	DRD4
		NOS3	TNFSF13B	REST	TXNIP		NFKB1	KRT10	PPARG			PLG	C1QTNF3				ZEB2	MAPK1	CAST	DDAH1	CFLAR	PDIA3	HPCA		IL23A	GREM1	MTOR	UBC	FLT3
		PRKG1	VSIG4	SMAD3			PDIA3	LTA	RBP4			PTEN	CAPN3					MTOR	CCL21	DDAH2	CYBB	PRKCA	IL18		MLLT4	GSK3B	NEDD4l		GLUL
		SERPINE1		SOD1			RIPK1	NCOA3	RELA			PTS	CARKD					PLAU	CCR7	DFFA	CYP24A1	RAC1	IL1A		MSN	HES1	PDIA3		GSN
				TM7SF2			S100A9	NFKB1	RIPK1			REST	CAV1					PTK2B	CD1C	DHCR24	CYP27B1	RPS6KB1	IL1B		NPS	IL7	PTEN		HDAC2
				TNFRSF1A			SERPINE1	PDIA3	TGFB1			SPR	CCL5					SMAD3	CD1D	DNMT1	DNMT1	SRF	IL1RN		PC	MAPK14	SOD1		MAPK1
				TNFSF10			SLIT2	S1PR3	TNF			TM7SF2	CSF3					SMAD7	CDC42	DNMT3l	DNMT3A	TGFA	IL6		RAG1	MMP15	TGFB1		NOTCH1
				VDR			SREBF2	SAG	TRAF6				CYP27B1					TGFB1	CDK1	DRD3	DRD1	THBS1	IL6R		RAP1B	MMP26	TLR2		NT5C
							TNF	SELE	VAV1				DDIT3					THPO	CDK4	DRD4	DRD3	TIAM1	JUN		VDR	NFATC4			NT5E
							ZNF580	SELP					DRD3					TNFSF4	CDK6	EDN1	DRD4	VTN	MAPK1			NOG			NT5M
								SERPINC1					DVL1						CDKN2A	FGF1	DRD5		MCOLN3			PDIA3			PROC
								SOSTDC1					ECD						CFL1	GRB2	EDN3		MKKS			PIN1			RAN
								TIMP2					EDN1						CFLAR	IGF1	FLT1		PDIA3			PPP1CC			REST
								TNF					EDN3						CREB1	IGF2	FRS2		PTK2B			PRKCH			SDS
								TNFAIP3					ETS1						CTNNB1	IL2RA	GPR124		RELA			PTGER2			SMAD3
								TRAF6					F7						CYP27B1	INSR	GSN		RPS6KB1			PTGER4			SOD1
													FABP1						DHCR24	MAP2K1	HDAC2		STAT3			PTK2B			TGFB1
													FADS1						DNMT1	MAPK1	IDO1					WNT2			TJP2
													FAM132B						DRD3	MCOLN3	INSR					WNT3			TNFRSF11A
													FASN						E2F1	MT-CYB	MAPK1					WNT9B			TNFSF11
													FGF2						EDN1	NEDD4l	NEDD4l								VDR
													FOXA1						EIF4EBP1	NFKB1	NF2								
													FOXO3						ELAVL1	OCLN	NOTCH1								
													FYN						EZH2	PARN	PALM								
													FZD6						F3	PDIA3	PCBP4								
													GCM2						FGF1	PPARG	PDIA3								
													GFI1						FLT1	PTK2B	PLAT								
													GPX1						FRS2	PTPN6	PLCB2								
													GRB10						FSCN1	RAG1	PLG								
													GRB14						FUT7	RIPK1	PROC								
													HAND1						FYN	RPS6KB1	PTEN								
													HCLS1						GLUL	SMAD2	PTPN1								
													HDAC1						GPR29	SOD2	PTS								
													HEY1						GRB2	SREBF2	RAG1								
													HEY2						GSN	TANK	SMAD2								
													ID1						HDAC2	TNFRSF1A	SMAD3								
													IGF1						HRAS	TNFSF10	SOD2								
													IGF2						HSPB8	TRAF6	TACSTD2								
													INSR						IGF1		TGFB1								
													IRS1						IGF2		TGFBR1								
													KLF4						IL23A		TLR2								
													KNG1						INS		TNFSF10								
													L3MBTL1						INSR		TNFSF4								
													LAMP2						IRS1		VDR								
													MAPK7						ITGB3		ZEB2								
													MTA1						KL										
													NAMPT						MDM2										
													NCOA2						MTOR										
													NFKB1						MYD88										
													NME1						MYLK										
													NR1D1						NOTCH1										
													NR1D2						PARN										
													NR1H3						PDIA3										
													PDK2						PIK3CA										
													PDPK1						PPARG										
													PER2						PTK2B										
													PIAS2						PTPN1										
													PIP4K2A						PTPN6										
													PPARA						RAG2										
													PPARG						RELA										
													PPARGC1A						RELB										
													PRDM16						RIPK1										
													PRKAB2						RPS6KB										
													PRKCA						RPS6KB1										
													PRKCE						S100A9										
													PTPRJ						SERPINE1										
													RORA						SLIT2										
													RPS6KB1						SMAD2										
													S100A8						SMAD3										
													S100A9						SOD2										
													SERPINA4						TCF7										
													SIRT1						TGFB1										
													SLC27A5						TGFBR1										
													SLIT2						TLR2										
													SPI1						TLR3										
													SREBF1						TNFRSF1A										
													SREBF2						TNFSF11										
													STUB1						TNFSF4										
													THBS1						TRAF6										
													TIAM1						TSC1										
													TNFAIP6						UBC										
													WLS						VDR										

### Relevance assessment of the vitamin D interactome in disease

**Figure 2:**
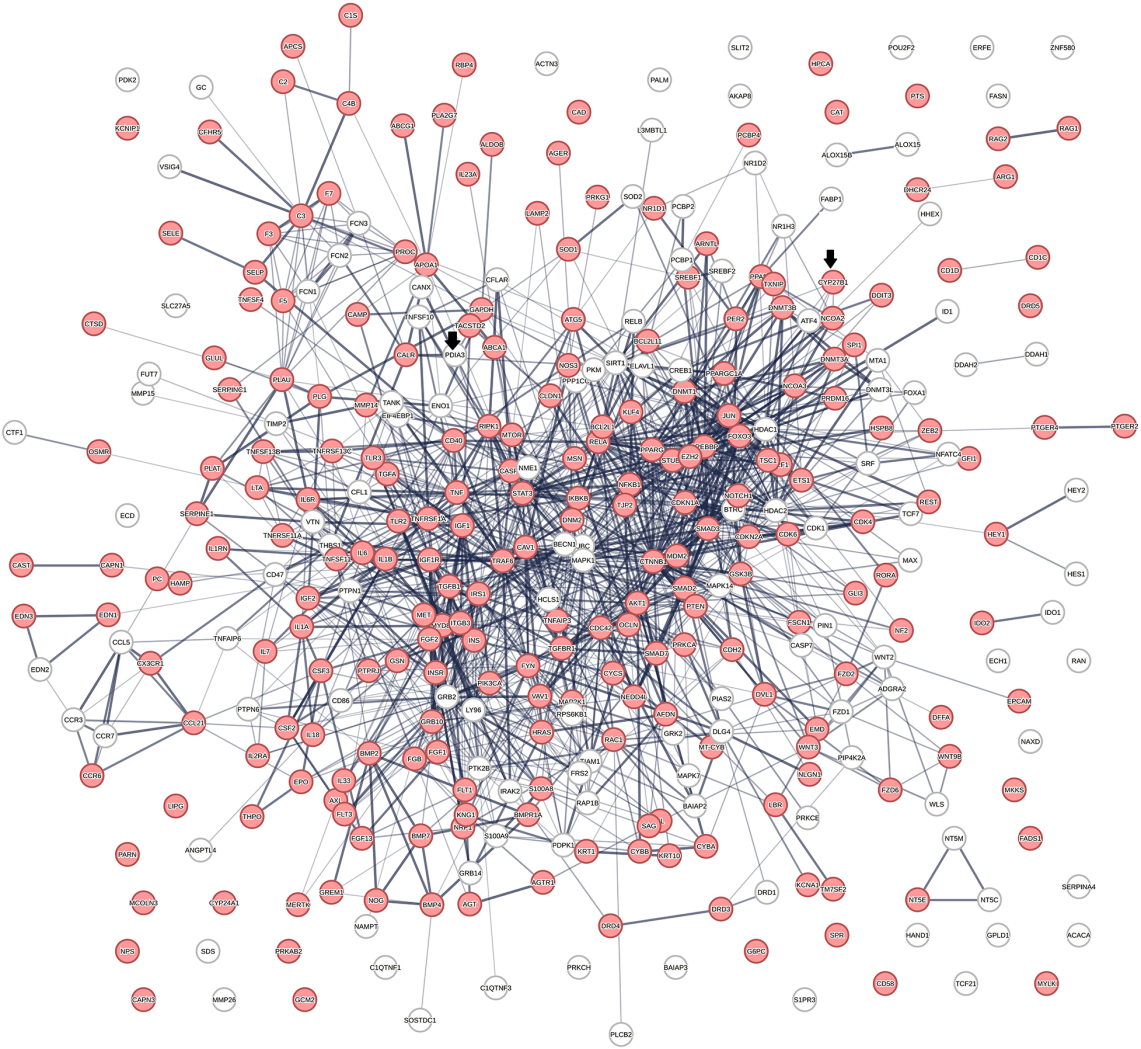
The physical interactions between 363 proteins captured in 29 vitamin D receptors containing swarm clusters. A total of 234 proteins (red) have known disease associations.

Figure 2 shows a protein network constructed from 363 proteins that were identified in 656 protein swarms described in Table 1. Further analysis to determine known disease associations of these proteins showed that 234 out of the 363 proteins in the vitamin D interactome have statistically significant disease associations (*p*-value 2.06 × 10^−33^). These findings establish the linkage between vitamin D signaling and the 4,821 diseases studied

Determination of the role of vitamin D signaling in 4,821 diseases and identification of cause–effect relationships associated with cardiovascular disease phenotypes

**Figure 3:**
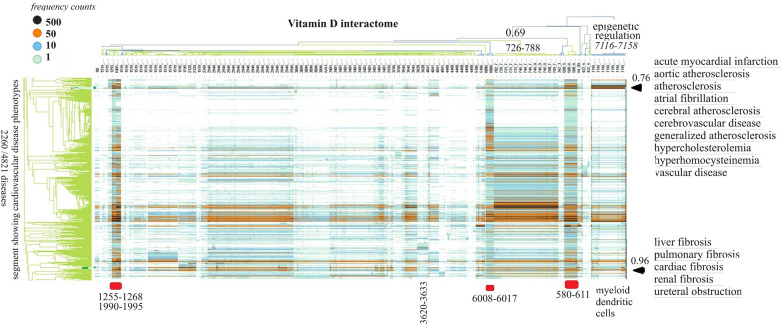
The horizontal dendrogram identifies the clusters of protein swarms that contain proteins engaged in similar functions. The vertical axis identifies the groups of diseases with similar cause–effect relationships. The hierarchical organization of protein swarms (CCSV > 0.69) shows that vitamin D-regulated processes are tightly coupled and subject to epigenetic regulation involving functions of myeloid dendritic cells (cluster 7,116–7,158).

Vitamin D receptor-associated protein swarms were labeled according to cluster membership and used for collecting co-citation frequencies associated with 4,821 diseases in a second round of Medline data mining. Hierarchical clustering of the resulting 4,821 × 656 similarity matrix followed by data visualization provided the heatmap shown in Figure 3.

Inspection of co-citation frequencies associated with disease and swarm clusters suggests that proteins in swarm clusters 580–611, 1,255–1,268, 1,990–1,995, and 6,008–6,017 (underscored in red) play a prominent role in 2,260 diseases. Furthermore, the dominant position of swarm cluster 7,117–7,158, in the hierarchical organization of protein swarms, indicates that vitamin D-mediated signaling is subject to epigenetic regulation involving myeloid dendritic cells (80, 81). Protein network construction using proteins in clusters 7,116–7,158 followed by functional analysis identified that protein interactions in these clusters regulate nitric oxide signaling (82), redox signaling (83), DNA methylation (84), and histone deacetylation (85). Similarly, the evaluation of *cause–effect* relationship similarities between diseases revealed that atherosclerosis, acute myocardial infarction, atrial fibrillation, cerebrovascular disease, hypercholesterolemia, hyperhomocysteinemia, and vascular disease are subject to similar regulatory mechanisms involving swarm clusters 580–611, 1,255–1,268, 1,990–1,995, 6,008–6,017, and 7,116–7,158 (CCSV > 0.76) (29).

### Vitamin D signaling in cardiovascular diseases

The effects of vitamin D signaling in cardiovascular diseases were further examined in three separate categories: (1) genomic signaling, (2) a mixture of genomic and non-genomic, and (3) non-genomic signaling.

#### Vitamin D genomic signaling

The initial focus was to determine functions regulated by proteins in swarms 7,117–7,158, 580–611, 1,255–1,268, 1,990–1,995, and 6,008–6,017 involved in 2,260 diseases containing the nuclear vitamin D receptor. Network construction using proteins in these clusters and retrieval of 10,000 publications with statistically significant protein network overlaps identified cardiovascular disease-associated functions regulated by the nuclear vitamin D receptor. Network overlaps between these functions are shown in Table 2. The results indicate that vitamin D's genomic signaling regulates protein–protein interactions involved in atherosclerosis, efferocytosis (B) (86, 87), macro-autophagy (C) (88), macrophage autophagy (D) (89, 90), macrophage polarization (E) (91, 92), cellular senescence (F) (93), ER stress response (G) (94), tissue homeostasis (H) (95), innate immunity (I) (96). caveola-mediated endocytosis (J) (97), and apoptosis (K) (98).

**Table 2:** The interactions between proteins in swarm clusters 7,117–7,158, 580–611, 1,255–1,268, 1,990–1,995, and 6,008–6,017 and overlaps of protein networks associated with atherosclerosis (A), efferocytosis (B), macro-autophagy (C), macrophage autophagy (D), macrophage polarization (E), cellular senescence (F), ER stress response (G), tissue homeostasis (H), innate immunity (I), caveola-mediated endocytosis (J), and apoptosis (K).

Protein network overlaps of clusters 7,117–7,158, 580–611, 1,255–1,268, 1,990–1,995, and 6,008–6,017										
A	B	C	D	E	F	G	H	I	J	K
AKT1	**x**	**x**	**x**	**x**	**x**	**x**	**x**	**x**	**x**	**x**
ATG5	**x**	**x**	**x**	**x**	**x**	**x**		**x**		**x**
AXL	**x**	**x**								**x**
CDK4		**x**		**x**	**x**		**x**			**x**
CTNNB1		**x**		**x**	**x**	**x**	**x**		**x**	**x**
CYCS	**x**	**x**		**x**	**x**	**x**				**x**
CYP27B1/VDR		**x**							**x**	
DDAH1		**x**								
DDAH2		**x**								
DNMT3A		**x**		**x**	**x**		**x**			**x**
DNMT3B		**x**		**x**	**x**		**x**			**x**
DRD4		**x**								
GRB2		**x**		**x**	**x**	**x**	**x**	**x**	**x**	**x**
GSN		**x**								**x**
HDAC2		**x**		**x**	**x**	**x**	**x**			**x**
MAPK1		**x**		**x**	**x**	**x**	**x**	**x**		**x**
MTOR		**x**	**x**	**x**	**x**	**x**	**x**	**x**		**x**
NOTCH1		**x**		**x**	**x**	**x**			**x**	**x**
NRP1		**x**		**x**			**x**			**x**
NT5E	**x**	**x**								**x**
PTEN	**x**	**x**	**x**	**x**	**x**	**x**		**x**	**x**	**x**
REST		**x**								
SMAD3		**x**		**x**	**x**	**x**	**x**		**x**	**x**
SOD1		**x**			**x**	**x**			**x**	**x**
SOD2		**x**			**x**					**x**
TGFB1		**x**		**x**	**x**	**x**			**x**	**x**
TNFRSF1A	**x**	**x**		**x**	**x**	**x**	**x**		**x**	**x**
TNFSF10	**x**	**x**						**x**		**x**
TNFSF11		**x**		**x**	**x**		**x**	**x**		**x**

**Figure 4:**
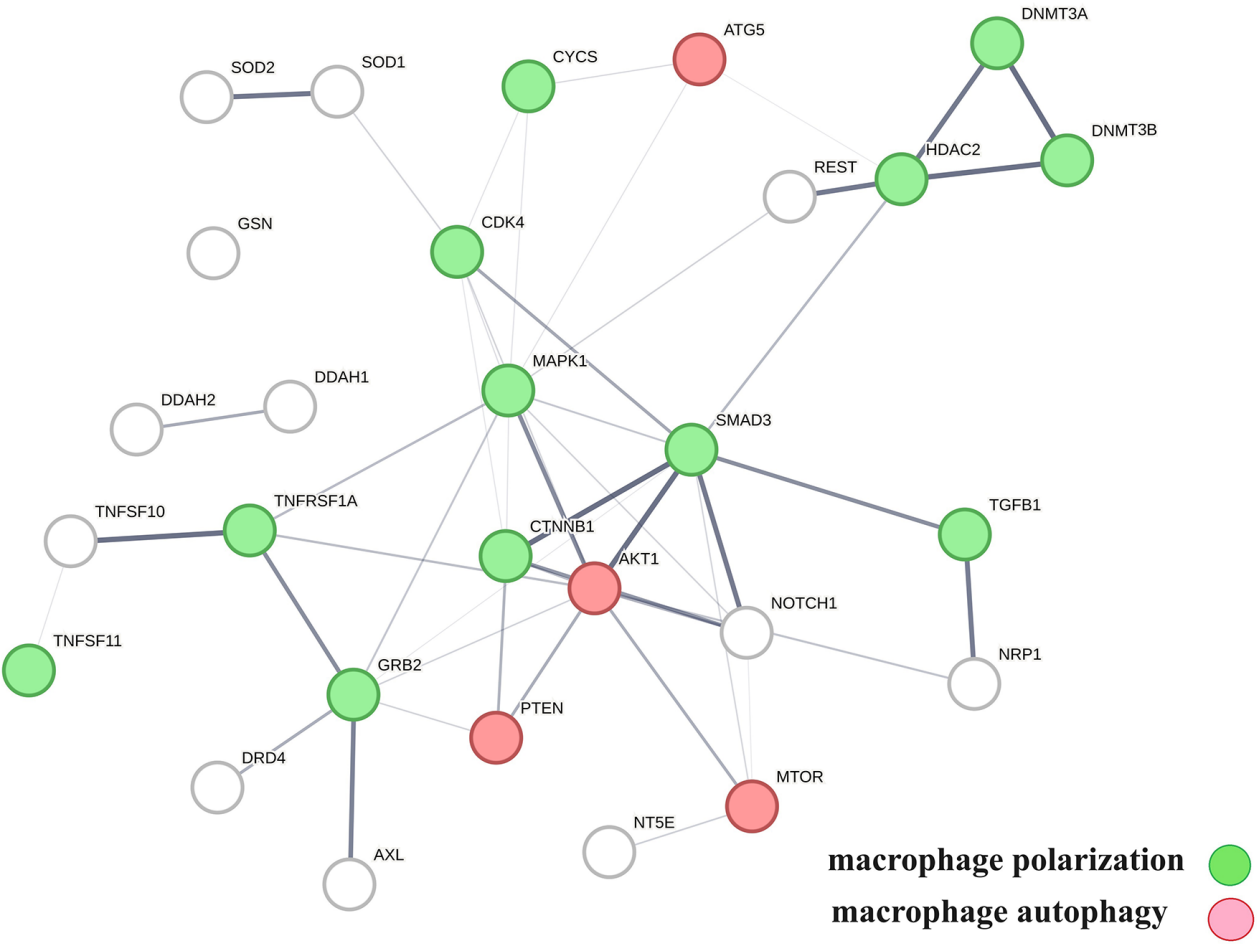
The physical interactions between proteins in swarm clusters 7,117–7,158, 580–611, 1,255–1,268, 1,990–1,995, and 6,008–6,017, highlighting the overlaps between protein networks associated with atherosclerosis (no color), macrophage polarization (green), and macrophage autophagy (red). Proteins ATG5, AKT1, and PTEN are involved in the regulation of all functions.

A protein network-centered view of results shown in Table 2 demonstrates that genomic signaling of vitamin D regulates at least 10 different functions involved in atherosclerosis (99–102) (Figure 4). Moreover, extensive protein network overlaps between these functions suggest that vitamin D's genomic signaling modulates a monitoring scheme that responds to nitric oxide signaling (DDAH1, DDAH2) (103), redox signaling (SOD1, SOD2, PDIA3) (104), environmental signals (DNMT3A, DNMT3B) (105, 106), histone acetylation (HDAC2) (107), inflammatory signals (TNFRSF1A, TNFSF10) (108), growth factor signaling (TGFB1, GRB2, NRP1) (109–111), mitochondrial status (CYS) (112), metabolic changes (MTOR) (113), and calcium signaling (GLSN) (114). Reflecting the importance of macrophages in atherosclerosis, Figure 4 shows that this regulatory scheme integrates macrophage polarization (115) and macrophage autophagy (88).

#### Vitamin D genomic and non-genomic signaling

To evaluate how the interaction between vitamin D's genomic and non-genomic signaling affects atherosclerosis, we focused on swarm cluster 726–788 (CCSV 0.99) containing the nuclear and non-nuclear vitamin D receptors. Protein network construction and retrieval of publications with statistically significant protein network overlaps identified cardiovascular disease-associated functions (Table 3).

**Table 3:** The interactions between proteins in swarm cluster 726–788 and overlaps of protein networks associated with atherosclerosis (A), efferocytosis (B), macro-autophagy (C), macrophage autophagy (D), macrophage polarization (E), cellular senescence (F), ER stress response (G), tissue homeostasis (H), innate immunity (I), caveola-mediated endocytosis (J), and apoptosis (K).

A	B	C	D	E	F	G	H	I	J	K
AGER	x	x		x	x	x		x		X
AGTR1		x		x	x	x		x		
APOA1	x	x		x		x		x	x	
BECN1	x	x	x	x	x	x		x		X
BMP2		x		x	x				x	
CAPN1		x				x				X
CASP8	x	x		x	x	x		x		X
CAV1		x		x	x			x	x	X
CCL5	x	x	x	x	x	x		x	x	X
CD40	x	x		x		x		x		X
CD58				x				x		X
CD86	x	x		x	x			x		X
CFL1		x						x		X
CYP27B1					x				x	
EDN1		x		x	x	x		x	x	X
FGF1		x		x		x				X
FOXO3		x		x	x	x	x	x	x	X
GRB2		x		x				x	x	X
IGF1	x	x		x	x	x	x	x		X
IKBKB	x	x		x	x	x		x		X
IL18	x	x	x	x	x	x	x	x	x	X
IL1B	x	x	x	x	x	x	x	x	x	X
IL1RN		x		x	x	x		x		
IL2RA		x		x	x	x	x	x		
IL33	x	x	x	x	x	x	x	x		X
ITGB3	x	x		x	x			x		X
LTA	x			x				x		X
NFKB1	x	x	x		x	x		x	x	X
PDIA3		x		x	x	x		x		
PPARG	x	x		x	x	x	x	x	x	X
RBP4		x								
RELA	x	x		x	x	x	x	x	x	X
RIPK1	x	x		x	x	x	x	x		X
S100A9		x						x		
S1PR3				x						
SAG		x				x		x		
SELE		x		x	x	x		x	x	X
SELP	x	x		x	x	x		x		X
SERPINC1		x		x	x	x		x		
SERPINE1		x					x	x	x	X
SLIT2					x					
SREBF2	x	x		x		x		x		
TGFB1	x	x	x	x	x	x	x	x	x	X
TGFBR1		x		x	x			x	x	X
TIMP2		x		x	x	x		x		X
TNF	x	x	x	x	x	x	x	x	x	X
TNFAIP3		x		x	x		x	x		
TRAF6	x	x	x	x	x	x	x	x	x	X
VAV1	x	x				x	x			
ZNF580										

**Figure 5:**
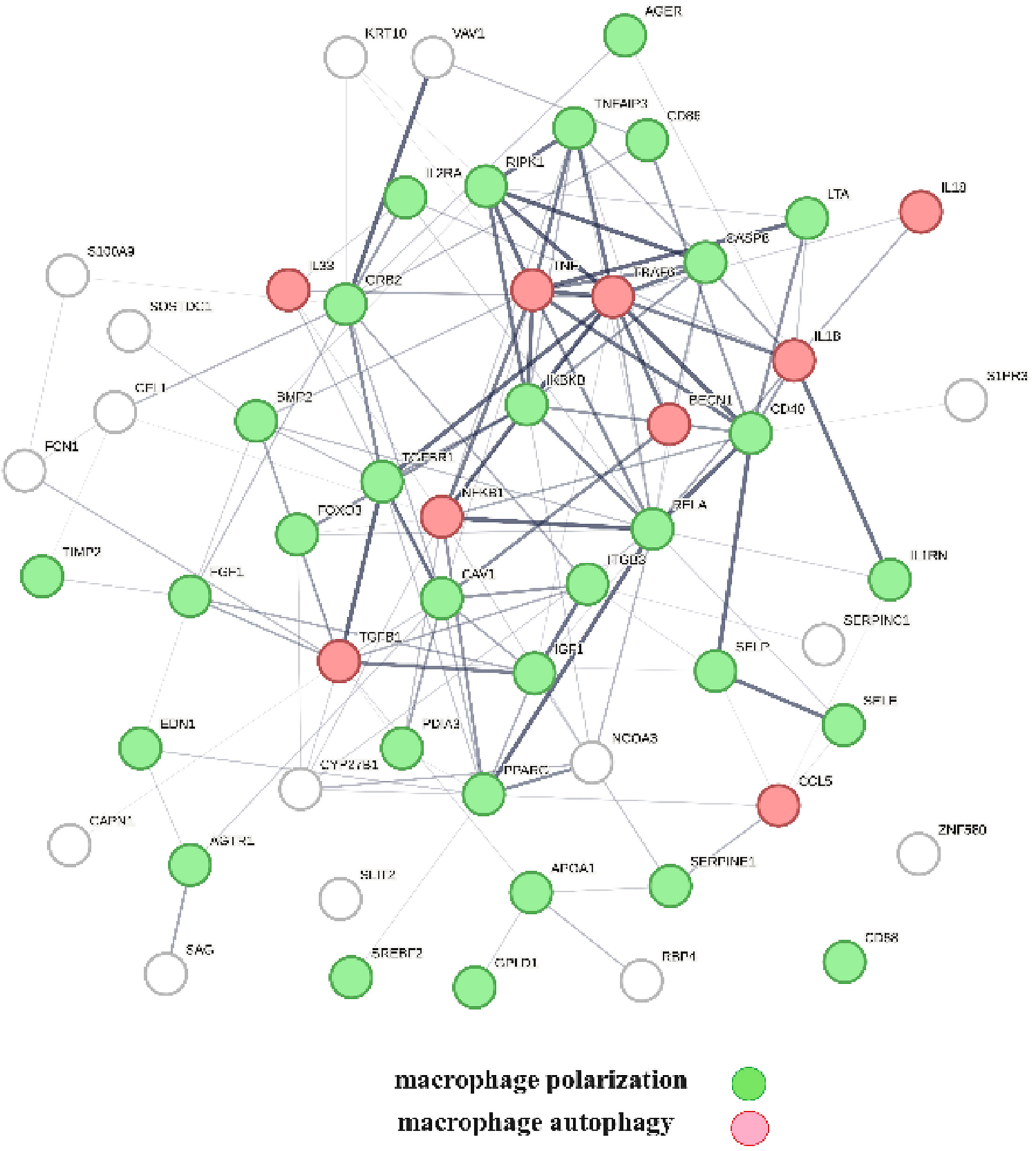
The protein interactions regulating vitamin D's genomic and non-genomic signaling in atherosclerosis. Proteins colored in green regulate macrophage polarization, and proteins colored in red regulate macrophage autophagy.

A protein network-centered view of Table 3 is shown in Figure 5. It highlights the interactions between proteins regulating macrophage polarization and macrophage autophagy in atherosclerosis (21). Specifically, the proteins colored in green regulate macrophage polarization, and the proteins highlighted in red are constituents of the NF-κB complex, which regulates inflammatory responses (116), autophagy, and macrophage autophagy (117). IL18 and TRAF6 nodes in the macrophage autophagy network regulate inflammatory processes and plaque instability in atherosclerosis (118, 119). Thus, this indicates that a mix of genomic and non-genomic signaling is involved in the regulation of atherosclerotic plaque stability.

#### Vitamin D non-genomic signaling

The first step prior to conducting cause–effect analysis was to identify clusters that only contained the non-nuclear vitamin D receptors. Specifically, there are clusters 3,780–788, 1,795–1,806, 3,620–3,633, 3,648–3,663, 3,766–3,775, 4,460–4,495, and 5,106–5,116. The next step was to evaluate if proteins in these clusters could physically interact with vitamin D's non-genomic receptor, i.e., PDIA3. This analysis identified that PDIA3 can physically interact with AKT1, BAIAP2, ENO1, GAPDH, GSK3B, MTOR, NFKB1, PCBP2, PCBP2, PDIA3, PPARG, SOD1, STAT3, and TGFB1. Protein network construction using this group of proteins and retrieval of publications with statistically significant network overlap identified that the interactions between these proteins modulate PI3K/AKT/mTOR signaling and that the natural product *rutin*, targeting this regulatory scheme, displays a U-shaped (non-linear) dose–response relationship in cardiomyocytes (120).

The involvement of the PDIA3 interactome in atherosclerosis was further assessed by identifying potential network–network interactions regulated by proteins in swarm cluster 3,620–3,633 (CCSV0.99) containing PDIA3 and proteins expressed in all tissues. Protein network construction using proteins in 3,620–3,633 clusters and retrieval of publications with statistically significant protein network overlaps identified cardiovascular disease-associated functions shown in Table 4. In contrast to the observations in Tables 2, 3, the absence of proteins regulating macrophage autophagy in Table 4 suggests that vitamin D's genomic and non-genomic signaling play different roles in regulating autophagy.

**Table 4:** The interactions between proteins in swarm cluster 3,620–3,633 and overlaps of protein networks associated with atherosclerosis (A), efferocytosis (B), macro-autophagy (C), macrophage autophagy (D), macrophage polarization (E), cellular senescence (F), ER stress response (G), tissue homeostasis (H), innate immunity (I), caveola-mediated endocytosis (J), and apoptosis (K).

3,620–3,633	A	B	C	D	E	F	G	H	I	J	K
ABCA1	x	x	x		x	x			x	x	x
AKT1	x	x	x		x	x	x	x	x	x	x
BAIAP2			x								x
CASP8	x	x	x			x	x		x	x	x
DLG4	x		x			x		x			x
FGF2	x	x	x			x	x	x	x	x	x
FZD2	x										x
MDM2	x		x			x	x	x		x	x
MERTK	x	x	x		x	x		x	x		x
MMP14	x		x			x	x	x	x	x	x
PDIA3			x				x				x
PRKCA	x		x		x	x	x		x	x	x
RAC1	x	x	x			x					x
RPS6KB1	x	x	x			x	x		x	x	x
SRF	x		x			x					x
TGFA	x		x		x	x	x		x		x
THBS1	x	x	x		x	x	x	x	x		x
TIAM1	x		x			x					x
VTN	x	x	x			x			x		x

**Figure 6:**
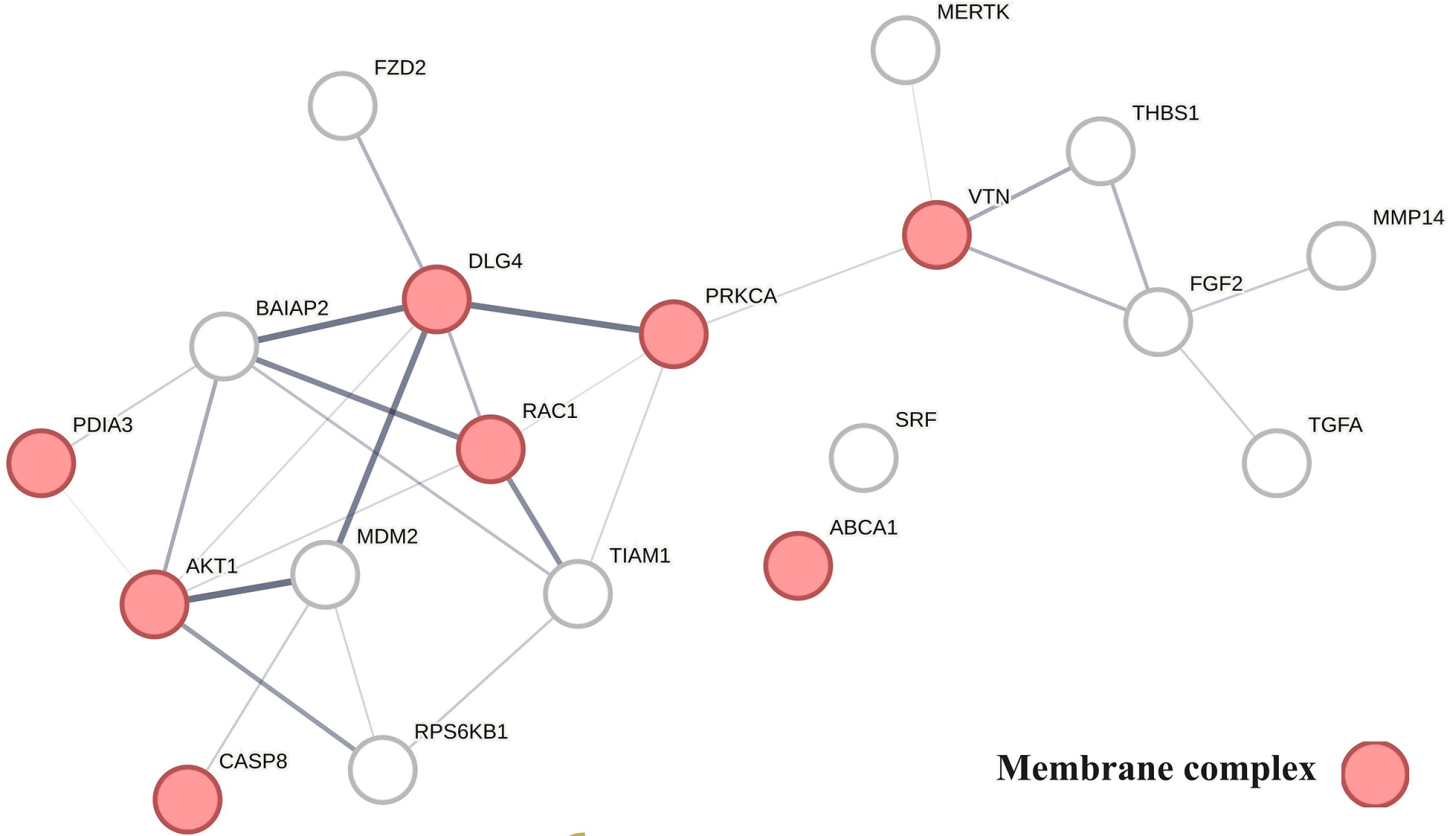
The physical interactions between proteins regulating vitamin D's non-genomic pharmacology in atherosclerosis.

Figure 6 provides a protein network-centered view of Table 4 and shows that proteins regulating vitamin D's non-genomic pharmacology can physically interact and affect atherosclerosis by regulating the balance between autophagy (121), apoptosis (122), endoplasmic reticulum stress response (94), cellular senescence (123, 124), and tissue homeostasis (125).

A key finding based on analysis of protein interactions in this network is that PDIA3 interactome (Figure 6) plays a key role in atherosclerosis. For example, interactions of PDIA3 with AKT1 (126) and interactions of AKT1with TIAM1 regulate the expression of the multicellular protein osteopontin (127, 128), which is involved in generating the atheromatous pathology of atherosclerosis (129). Furthermore, interactions of PDIA3 with brain-specific angiogenesis inhibitor 1-associated protein 2 (BAIAP2; a.k.a. IRSP53) regulate apoptotic cell clearance (130), plasma membrane shape homeostasis, and endocytosis. Mutations in tyrosine kinase-defective Mertk receptor MERTK decreases macrophage autophagy and increases the size of necrotic plaque in atherosclerosis (131, 132). Interactions of BAIAP2 with TIAM1, RAC1, and DLG4 regulate filopodia extension via actin polymerization (133) and regulate LDL metabolism by macrophages (134, 135). In addition, interactions of BAIAP2 with the WAVE regulatory complex (136, 137) control highly dynamic processes involved in cholesterol clearance, embryogenesis, neuron morphogenesis and plasticity, immune cell activation, chemotaxis, fibrosis, and cancer invasion and metastasis (138–141).

The finding that PDIA3 interactome regulates multiple reciprocal feedback loops is supported by observations showing that calcitriol, in combination with p53 aka TP53 regulates the expression of MDM2 (142), occupying a central position as shown in Figure 6. Further, the regulation of MDM2 expression creates an autoregulatory feedback loop wherein the expression of MDM2 is coregulated by TP53 protein levels and MDM2 protein levels decrease the ability of TP53 to function as a positive transcription factor for MDM2 (143). Since the regulation of TP53 expression regulates the inactivation of SREBP2, this feedback loop regulates the activities of mevalonate and PCSK9 pathways, which are key players in atherosclerosis (144, 145). Similarly, reciprocal regulation of TP53 expression and nitric oxide levels via regulation of expression of nitric oxide synthases (NOS2, NOS3) (146, 147) and methylation and demethylation of arginine (swarm cluster 7,116–7,158) creates additional feedback loops (148, 149). Closing these feedback loops, MDM2 regulates the expression of the nuclear vitamin D receptor (150, 151), and calcitriol, in coordination with TGFB1, regulates PDIA3 expression (152, 153). Thus, these two feedback loops regulate the ratio of nuclear and non-nuclear expression of vitamin D receptors. This finding suggests that this form of regulation can fine-tune the ratio between genomic and non-genomic signaling of vitamin D and can operate independently from the binding affinities of calcitriol. Since vitamin D effects are antifibrotic whereas PDIA3 effects are profibrotic, these feedback loops may be involved in balancing processes involved in tissue homeostasis.

Adding further complexity to the TP53-mediated regulatory scheme is that ER stress-induced posttranslational modifications of P53 affect the sensitivity of MDM2 and TP53 feedback loops (154, 155). The impact of these feedback loops on the outcomes of pharmacological studies using vitamin D is manifested in clinical and preclinical observations showing U-shaped dose–response relationships (156–160).

## Discussion

This study provides deeper insights into the role of vitamin D in cardiovascular disease development. The use of a spectral clustering method for identifying vitamin D-regulated processes in 4,821 diseases provides a global effect positioning system that can be used to determine *cause–effect* relationships in atherosclerosis. Examination of vitamin D-associated genomic and non-genomic pharmacologies using this positioning system revealed origins of non-linear dose–response relationships observed in preclinical and clinical studies with vitamin D. In addition, it also identified functional relationships between multiple reciprocal feedback loops that sometimes regulate the opposing functions activated by nuclear and membrane-associated vitamin D receptors. Among the processes of relevance for the treatment of cardiovascular diseases are p53-mediated regulation of atherosclerotic plaque stability and macrophage autophagy, which are known to safeguard the plasticity of macrophages in tissue homeostasis. We have used an unsupervised machine learning approach for deciphering these complex *cause–effect* relationships and used protein network analysis for identifying interactions between protein networks that regulated the functions that are of relevance in atherosclerosis. The initial step was aimed at establishing a global effect positioning system (Table 1) using information theory-based methodology to allow for an unbiased analysis of vitamin D pharmacology in diseases. Information flow-based *cause–effect* analysis of 4,821 diseases led to the identification of the vitamin D interactome (Figure 2) comprising 363 proteins, where 64% of proteins were found to have highly statistically significant (*p*-value = 2.06 × 10^−33^) disease associations. This observation establishes the value of our protein swarm-based framework for investigating *cause–effect* relationships in protein–protein interaction (PPI) networks and complements previous work reported by Silverbush and Sharan (161) who used drug response and cancer genomic data to orient the human PPI network.

Examination of the role of the vitamin D interactome in 4,821 diseases provided two key findings: (1) vitamin D-regulated processes are involved in over 2,000 diseases including cardiovascular disease, and (2) all functions regulated by vitamin D are subject to epigenetic regulation (Figure 3). Epigenetic regulation of atherosclerosis is well established (162), and our finding of the involvement of vitamin D signaling contributes to the development of diagnostics or therapeutic agents in atherosclerosis. Also depicted in Figure 3 is the observation that cardiovascular diseases including atherosclerosis are affected by PPI in swarms 580–611, 1,255–1,268, 1,990–1,995, 6,008–6,017, and 7,116–7,158.

The effects of vitamin D signaling in cardiovascular diseases were further examined as three separate categories: (1) genomic signaling mediated by PPI in swarms 580–611, 1,255–1,268, 1,990–1,995, 6,008–6,017, and 7,116–7,158 (Table 2), (2) mixture of genomic and non-genomic signaling mediated by PPI in swarms 726–788 (Table 3), and (3) non-genomic signaling mediated by PPI in swarms 3,620–3,633 (Table 4). Tables 2–4 show the results of evidence-based *cause–effect* analysis using network overlaps of proteins in the three swarm categories with PPI networks of atherosclerosis and the 10 biological functions affected by aging and involved in atherosclerosis. The significance of vitamin D's genomic, mixed genomic, and non-genomic signaling in atherosclerosis related to the 10 biological functions associated with atherosclerosis (Figures 4–6) was determined by measuring the extent of network overlap, ability to engage in physical interactions, and centrality of shared network nodes. Hence, the main inference from the results shown in Tables 2, 3 and Figures 4, 5 is that genomic and mixed genomic signaling by vitamin D modulates the 10 important biological functions associated with atherosclerosis. Additionally, examination of network overlaps between these 10 biological functions indicated that they are part of an integrated regulatory scheme that includes macrophage polarization and macrophage autophagy as central elements.

The effects of vitamin D's non-genomic signaling are summarized in Table 4 and Figure 6. The absence of overlap between proteins in swarm cluster 3,620–3,633 and macrophage autophagy in combination with the observation that these clusters are enriched with proteins involved in fibrosis suggests that non-genomic signaling by vitamin D plays an important role in tissue homeostasis.

The identification of protein network–network interactions described above allows the generation of a comprehensive view detailing how interactions between the 10 biological functions affect atherosclerosis.

A critical factor in atherosclerosis-associated mortality is plaque stability (163), where stable plaque forms carry a low risk of sudden mortality while rupture of unstable forms causes myocardial infarction and stroke. Our analysis (Tables 2, 3; Figures 4, 5) indicates that vitamin D signaling plays a key role in balancing plaque stability (164) by regulating macrophage autophagy (90) and macrophage polarization, a key determinant of the M1/M2 ratio, where macrophages with M1-like characteristics are associated with the inflammatory stages of atherosclerosis while macrophages with M2-like characteristics have anti-inflammatory characteristics and promote plaque regression (91, 165). Declining macrophage autophagy increases macrophage apoptosis, decreases macrophage efferocytosis, increases oxidative stress, and destabilizes plaque (132, 166).

A unique property of macrophages is that they can directly generate calcitriol, which is the most active form of vitamin D, in response to a variety of input signals (167). Calcitriol, in turn, activates macrophage autophagy and shifts the macrophage polarization toward anti-inflammatory M2-like macrophages (168, 169). Thus, by shifting macrophage polarization toward M2 (170), calcitriol can decrease inflammation (171) and increase the clearance of cellular debris by efferocytosis (172).

Additionally, of relevance to the development of atherosclerosis is aging. Aging shifts macrophage polarization toward M1 phenotypes (173), increases cellular senescence (174) and inflammation (175), and decreases macrophage functions (176). Calcitriol can counter aging-induced acceleration of atherosclerosis at the plaque initiation (177–179), progression (179, 180), rupture (181), regression (182–184), and healing stages of atherosclerosis (179, 185). Accordingly, shifting macrophage ratios from M1 to M2 controls plaque size and stability (170). For example, low shear stress or high endoplasmic reticulum negatively affects autophagy, and shifting macrophage polarization toward inflammatory M1 accelerates atherosclerosis (186–190). Vitamin D deficiency exacerbates this development by increasing M1 macrophage populations and decreasing autophagy (115, 191). Calcitriol administration can reverse this trend by shifting macrophage populations toward M2 phenotypes, which increases macrophage autophagy (192–195).

However, an important finding of our investigation is that the over-enrichment of M2 macrophage populations can result in excessive fibrosis. In other words, vitamin D's non-genomic signaling via PDIA3 at higher calcitriol concentrations promotes fibrosis, leading potentially to plaque destabilization (196). Maintenance of the delicate balance between M1 and M2 macrophage subpopulations is critical as it affects tissue homeostasis. Experimental data suggest that too much or too little on either side of the equation results in detrimental outcomes (197). This balance can be affected in multiple ways. For example, the involvement of immune responses in this delicate balancing act is implicated by observations that cathelicidins produced by vitamin D's genomic response to infections activate autophagy and shift macrophage populations toward M2 (198). In addition, the identification of regulatory schemes clearly establishes that vitamin D regulates multiple feedback loops (76, 199–203) involved in tissue homeostasis, which are subject to additional regulation by nitric oxide (204) and redox signaling (205). Evidence of these types of non-linear (U-shaped) *cause–effect* relationships/dose responses, expected due to opposing pharmacologies, is indeed observed in numerous preclinical and clinical studies with vitamin D [see references (156–160)].

## Strength and limitation

The dynamics of protein–protein interactions impart functional diversity to proteomes and balance functions separating health and diseases. From a whole organism perspective, single-cell approaches do not provide the system-level insight that is needed to understand how interactions between proteomes at tissue levels affect the progression of a disease or the responses of organisms to treatments (206). To generate the required insights, integrated approaches are needed that can determine relationships between large heterogeneous, unstructured, and noisy data stored in different databases. By taking advantage of the modular designs of proteomes and principles governing information transfers in integrated systems (207, 208), protein swarm-based cause–effect analysis delivers unbiased insight into how interactions between tissue- and cell-associated proteomes regulate the physiological and pathological states of organisms.

“Swarm Intelligence” based analysis reveals cause-effect relationship between the many variables involved and provides understanding that is very difficult to ascertain with other means.

The reach of the current vitamin D-centered cause–effect analysis is limited by its focus on determining the system pharmacology of calcitriol. Therefore, our study neglects the potential contributions of calcidiol or the various vitamin D metabolites. Furthermore, technical constraints limit the resolution of network–network interactions by setting upper limits to the number of protein swarms used for sampling tissue proteomes. Lastly, the limiting capability of machine learning approaches in the biological cause–effect analysis is the scarcity of data on the concentration time dependency of dynamic cause–effect relationships at a system-wide scale.

## Summary

Using a global framework for proteomics-based *cause–effect* analysis, our analysis identified a broad vitamin D-regulated scheme involved in tissue homeostasis of over 2,000 diseases. By providing a perspective on how interactions between vitamin D's genomic and non-genomic actions, nitric oxide and redox signaling, macrophage autophagy, macrophage polarization, and p53 signaling shape tissue homeostasis, our analysis addresses a long-standing enigma presented by observations that vitamin D supplementation, despite projected benefits, produces little benefits in clinical studies (209). Recognizing the importance of p53, nitric oxide, and redox signaling in vitamin D-regulated processes offers the opportunity to increase the efficacy of vitamin D supplementation in cardiovascular diseases. Toward this end, modalities integrating nitric oxide redox signaling and vitamin D-centered pharmacologies have already shown promising outcomes (210). Refinement of these approaches is expected to improve the treatment of cardiovascular diseases and increase the quality of life in aging (211).

## Data Availability

The original contributions presented in the study are included in the article/Supplementary Materials; further inquiries can be directed to the corresponding author.
